# Supplementary material for: Tunable PhenoCycler imaging of the murine pre-clinical tumour microenvironments
Source: Cell Biosci. 2024 Feb 4;14:19. doi: 10.1186/s13578-024-01199-4 (PMC10840224; doi:10.1186/s13578-024-01199-4)
Supplement: Supplementary file 1 — Supplementary Material 1: Immunofluorescence optimization of antibodies for murine FFPE staining [file 13578_2024_1199_MOESM1_ESM.docx]

**Tunable PhenoCycler Imaging of the Murine Pre-Clinical Tumour Microenvironments**

Madelyn J. Abraham, Christophe Goncalves, Paige McCallum, Vrinda Gupta, Samuel E. J. Preston, Fan Huang, Hsiang Chou, Natascha Gagnon, Nathalie A. Johnson, Wilson H. Miller Jr^*^, Koren K. Mann^*^, and Sonia V. del Rincon^*^

**SUPPLEMENTAL MATERIAL**

**
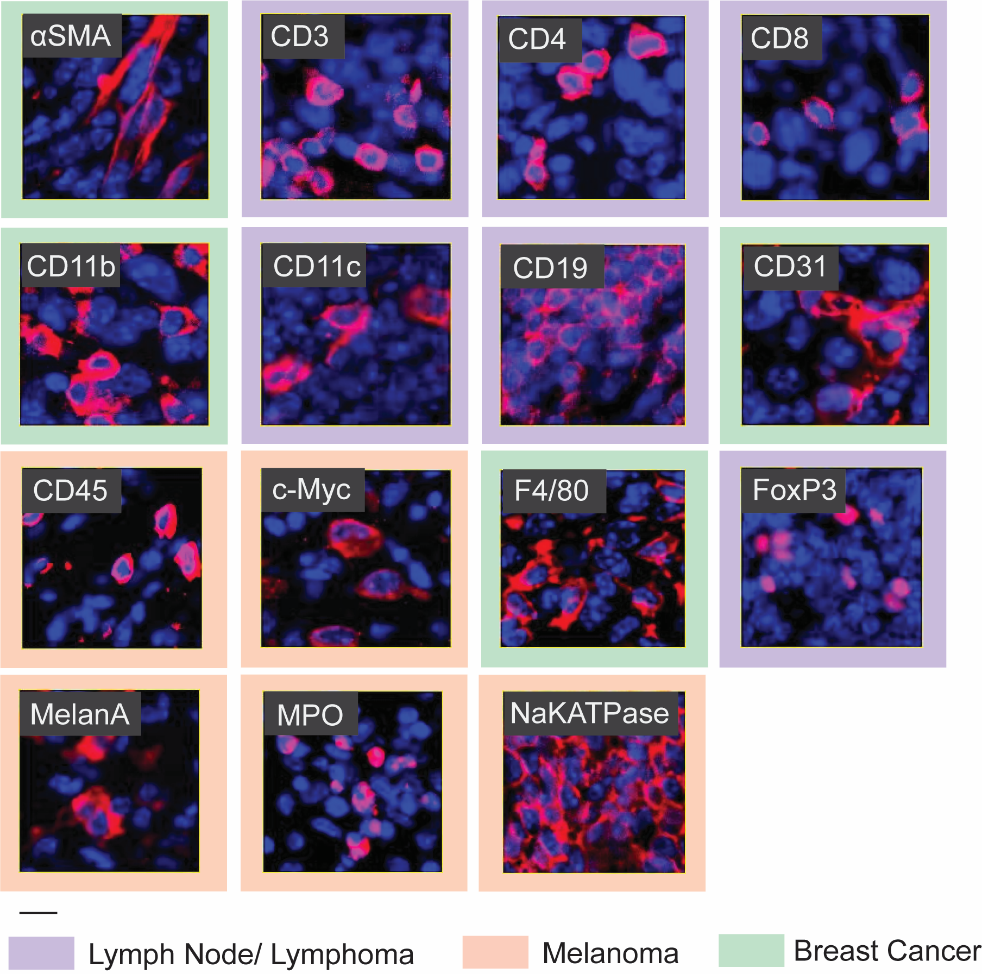
**

**Supplemental Figure 1: Immunofluorescence optimization of antibodies used to stain murine FFPE preserved tissues.**

Images showing optimized immunofluorescence staining of each antibody that was subsequently conjugated with an oligonucleotide barcode. In each image, DAPI is blue, and each individual marker is red. In each case, AF647-tagged secondary antibodies were used. The colour of the outer border indicates the tissue type imaged. Scale bar is 15 µM.
